# Paradoxical physiological responses to propranolol in a Rett syndrome patient: a case report

**DOI:** 10.1186/s12887-016-0734-3

**Published:** 2016-11-29

**Authors:** P. J. Santosh, L. Bell, K. Lievesley, J. Singh, F. Fiori

**Affiliations:** 1 Department of Child Psychiatry, Institute of Psychiatry, Psychology and Neuroscience, King’s College London, London, UK; 2 Centre for Interventional Paediatric Psychopharmacology and Rare Diseases, South London and Maudsley NHS Foundation Trust, London, UK

**Keywords:** Rett syndrome, MeCP2, Propranolol, Generalised anxiety disorder, Dysautonomia, Automatic monitoring of physiological parameters

## Abstract

**Background:**

Rett Syndrome (RTT), caused by a loss-of-function in the epigenetic modulator: X-linked methyl-CpG binding protein 2 (MeCP2), is a pervasive neurological disorder characterized by compromised brain functions, anxiety, severe mental retardation, language and learning disabilities, repetitive stereotyped hand movements and developmental regression. An imbalance in the sympathetic and the parasympathetic nervous system (dysautonomia) and the resulting autonomic storms is a frequent occurrence in patients with RTT. The prototypical beta blocker propranolol has been used to manage sympathetic hyperactivity in patients with RTT.

**Case presentation:**

A 13 year old girl with RTT was referred to the Centre for Interventional Paediatric Psychopharmacology and Rare Diseases (CIPPRD), South London and Maudsley NHS Foundation Trust. Her clinical picture included disordered breathing with concomitant hyperventilation and apnoea, epilepsy, scoliosis, no QT prolongation (QT/QTc [372/467 ms on automated electrocardiogram [ECG], but manually calculated to be 440 ms]), no cardiac abnormalities (PR interval: 104 ms, QRS duration: 78 ms), and generalised anxiety disorder (ICD-10-CM Diagnosis Code F41.1). She was also constipated and was fed via percutaneous endoscopic gastrostomy (PEG). To manage the dysautonomia, propranolol was given (5 mg and 10 mg) and in parallel her physiological parameters, including heart rate, skin temperature and skin transpiration, were monitored continuously for 24 h as she went about her activities of daily living. Whilst her skin temperature increased and skin transpiration decreased, unexpectedly there was a significant paradoxical increase in the patient’s average heart rate following propranolol treatment.

**Conclusion:**

Here, we present a unique case of a paradoxical increase in heart rate response following propranolol treatment for managing dysautonomia in a child with RTT. Further studies are warranted to better understand the underlying dysautonomia in patients with RTT and the impact this might have on treatment strategies in rare disorders such as RTT.

## Background

Ever since Dr Andreas Rett first observed the marked hand stereotypies (hand wringing) of two severely disabled young girls in a paediatric clinic waiting room in Vienna circa 1965, research into Rett Syndrome (RTT) has progressed at a significant rate. RTT is a neurodevelopmental disorder that manifests in young girls with an incidence of about 1: 10,000 female live births [1]. Although initial reports suggested exclusivity of RTT in females, due to the fact that in males the mutation was often embryonic lethal, males with milder mutations can survive and at present 57 cases of RTT have been reported in males [2]. The clinical picture presents with a constellation of neurological symptoms ranging from anxiety, replacement of meaningful hand movements with repetitive stereotypies, seizures, neurogenic apnoea, breathing abnormalities and a prominent delay or absence in speech [3] but also less prominent non-neurological pathologies such as osteopenia, scoliosis, gastrointestinal disorders, and a general retardation in growth [3]. RTT belongs to a larger group of neurodevelopmental disorders known as “synaptopathies” which are usually disorders with aberrations in synaptic function and/or morphology being implicated in the pathogenesis of the disease. At a cellular level, evidence has indicated that neurons in patients with RTT are smaller; more densely packed, reduced in length and complexity, and reduced in the number of dendritic spines [4]. This specific neuronal architecture might help to unravel the clinical picture of the disease and provide mechanistic insight into brainstem autonomic dysfunction in RTT. RTT has functional convergence with other neurodevelopmental disorders and is thought to share many of the altered molecular signalling pathways with Autism Spectrum Disorder (ASD). This partnership is underscored due to the fact that individuals with RTT were often diagnosed as having ASD. Indeed, about half of children with RTT also exhibit symptoms consistent with those of ASD [5].

Around 90% of cases of patients who exhibit classical RTT have loss-of-function mutations of the critical epigenetic modulator: X-linked methyl-CpG binding protein 2 (MeCP2) [6] that is purported to cause neurological dysfunction by specifically disrupting long gene expression within the brain [7]. Due to the chimeric expression of the mutant allele and the pattern of X-linked inactivation, RTT exhibits considerable pleiotropic heterogeneity and the clinical picture ranges from individuals who are high functioning, to those who are severely incapacitated. Individuals with RTT appear to develop normally up to 6–18 months of age. However, this is followed by a period of developmental stagnation and regression with significant loss of speech and social interaction. It was unclear why these symptoms manifest after this period of relative quiescence; however this quandary was deciphered recently when Chen and colleagues showed that MeCP2 adopts differing roles when recognising methylated DNA to regulate gene transcription in the mammalian CNS and this contributes to the delayed onset of RTT pathologies [8]. Several key cellular players have been implicated in propagating RTT pathology [9]; however the confounding results in the roles for some of the emerging players [10, 11] casts doubt on whether they may actually play a substantive role in the progression of RTT pathology. Research investigating the pathologies of RTT is still in its infancy and the clinical picture seems to be changing frequently. Recent evidence has shown that deep brain stimulation can improve the hippocampal memory deficits in a mouse model of RTT [12] whilst SUMOylation of MeCP2 was able to restore behavioural deficits in a mouse model of RTT [13]. How well these findings extrapolate to studies done in patients with RTT at present remains unclear.

Individuals with RTT might survive into adulthood; however their life expectancy is curtailed due to the higher frequency of sudden and unexpected death. Analysis of deaths from a British survey has shown that about 25% of all deaths in RTT are sudden and unexpected [14] and it has been surmised that the torsadogenic features in cardiac physiology that are intrinsic to certain individuals with RTT could explain some of these deaths [15]. Indeed, it has been shown that girls with RTT have clinically significant prolongations in the QTc interval and T-wave abnormalities in comparison to age-matched healthy controls [16].

In terms of syndrome progression and the development of dysautonomia, there is a scarcity within the evidence base providing syndrome progression from a dysautonomia perspective in patients with RTT. It is likely that post-translational modifications of long genes implicated in neuronal development [7, 17] regulate the functional and developmental versatility of MeCP2 in individuals with RTT. Recent evidence has shown that *MeCP2* ^*R306C*^ mutation prevents it from interacting with the NCoR/histone deacetylase 3 (HDAC3) complex, which causes impairments in social and cognitive functioning [18]. In the developing brain stem, this impairment will more than likely have downstream effects such as perturbations in excitatory [19] and inhibitory [20] pathways, which in turn will have knock-on effects on vital brain regions implicated in sympathetic and parasympathetic elements of the autonomic nervous system (ANS). Any imbalance will result in dysautonomia; however whilst, autonomic manifestations in RTT include anxiety, pupillary dilation [21], raised serotonin receptor binding in brain stem nuclei [22], low vagal tone with poor vagal response to hyperventilation [23], lower heart rate variability with prolonged QTcF [15] and uncontrolled albeit normal sympathetic tone due to minimal negative feedback of the parasympathetic system [24], due to the inherent heterogeneous plasticity of RTT particularly in terms of maturity-related brainstem functioning [25], these manifestations are more than likely to differ between individuals and so the precise trajectory in terms of the developmental progression of dysautonomia in RTT is at the moment unclear.

Given that dysautonomia is a cardinal feature of RTT, a logical treatment regimen would be for the introduction of a cardio-protective agent to dampen down the paroxysmal sympathetic hyperactivity that is frequently encountered in individuals with RTT. The prototypical beta-blocker propranolol is useful in this regard, as beta-adrenergic receptor blockade might also help to ameliorate the somatic manifestations of dysautonomia such as palpitations, tremor, diaphoresis and panic attacks which occur in up to 75% of cases in RTT [26]. Despite these findings, there is some circumspect for the role of beta blockers as an efficacious agent for managing dysautonomia in individuals with RTT. Findings from murine studies have indicated that acute treatment with propranolol does not prevent ventricular tachycardia in mice lacking MeCP2 that have an increased susceptibility to ventricular arrhythmias [27]. Moreover, chronic propranolol has no effect on the prolongation of the QTc interval and also failed to prevent cardiac arrhythmias in MeCP2 deficient mice [28].

Given the finding that acute or chronic treatment with propranolol might not be an effective therapy for managing dysautonomia in RTT and about 20% of sudden deaths in RTT being of cardiac origin, it is imperative to explore these findings in humans. No prior case reports have incorporated a psychiatric evaluation on the effectiveness of propranolol in managing dysautonomia in a child with RTT. Hitherto in this current report, we specifically used the prototypical beta-blocker propranolol to modify the dysautonomia in this patient with a view to ameliorate the concomitant features such as cardiac instability, diaphoresis and panic attacks.

## Case presentation

In November 2013, a 13 year-old girl of British origin, with a confirmed genetic and clinical diagnosis of Rett Syndrome (RTT) was referred to the Centre for Interventional Paediatric Psychopharmacology and Rare Diseases (CIPPRD), South London and Maudsley NHS Foundation Trust. She presented with disordered breathing with concomitant hyperventilation and apnoeas, epilepsy, scoliosis (has had previous spinal cord surgery), no QT prolongation (QT/QTc [372/467 ms on automated electrocardiogram [ECG], but manually calculated to be 440 ms]), no cardiac abnormalities (PR interval: 104 ms, QRS duration: 78 ms), and generalised anxiety disorder (ICD-10-CM Diagnosis Code F41.1). She was also constipated and was fed via percutaneous endoscopic gastrostomy (PEG). Her treatment regimen included lamotrigine (150 mg in the morning and 90 mg in the evening), clobazam as required (10 mg rescue medication for seizures), slow release melatonin (10 mg in the evening), Movicol (2–3 sachets a day), omeprazole (20 mg bid), L-carnitine supplements, calcium supplements and Senokot (as required). She also received topical treatment for a fungal skin infection.

Propranolol was newly administered (5 mg and 10 mg) and physiological parameters including heart rate, skin temperature and skin transpiration were monitored. This was following a clinical protocol routinely used in the CIPPRD, specifically the joint parent-clinician shared decision-making model ‘Effective Dosing with Minimum Side Effects’ (EDMS) strategy [29, 30]. The EDMS strategy aims to use the lowest possible dose of a medication to achieve adequate management of symptoms with minimal side effects. Initially, 5 mg propranolol was administered. At this dose, the patient did not experience discomfort and so in a joint shared clinical decision making process with the parents and clinicians, it was decided that the dose of propranolol should be increased to 10 mg. Following increase to 10 mg, the patient became distressed and so treatment was terminated.

In order to estimate the baseline, these parameters were also recorded in a treatment free period (24 h). The CIPPRD is experienced in using a variety of wearable technologies for the non-invasive physiological monitoring in patients and in this patient the Basis B1 wristband [31] was used for real-time monitoring that recorded the heart rate, skin temperature and skin transpiration continuously for 24 h as the teenager went about her activities of daily life such as attending school and when at home.

**Fig. 1:**
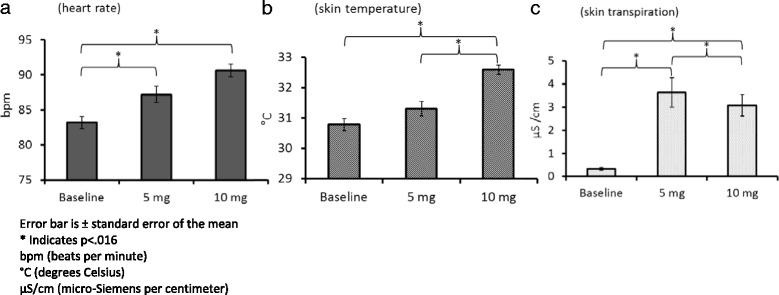
Effect of propranolol on heart rate (**a**), skin temperature (**b**) and skin transpiration (**c**)

Data were manually obtained from the plots on the device webpage. The recorded physiological parameters were divided into epochs of 30 min during the 24 h period for each dose of medication. For each epoch, the average value for all the physiological parameters was estimated using a 5 min interval. Three one way ANOVAs (one for each parameter) were performed. Unexpectedly there was a concomitant significant concentration dependent increase in heart rate from baseline (Fig. 1a) with the maximum heart rate measured at 10 mg (F_[1.806]_ = 17.010; *p* < .001; baseline versus 5 mg dose: t_[49]_ = −3.286; *p* < .016; baseline versus 10 mg dose: t_[49]_ = −6.649; *p* < .016, using Bonferroni Correction for multiple comparisons [Bfc]). The highest dose of propranolol (10 mg) paradoxically increased the heart rate by about 10 beats per minute and was associated with a significant worsening of night time sleep quality and distress reported by the parent, and consequently the treatment was discontinued. In parallel, there was a significant increase in the skin temperature (F_[1.683]_ = 32.986; *p* < .001; baseline versus 10 mg dose: t_[49]_ = −8.084; *p* < .016 Bfc; 5 mg dose versus 10 mg dose: t_[49]_ = −6.941; *p* < .016 Bfc) (Fig. 1b) and a significant decrease in her skin transpiration (F_[1.040]_ = 34.174; *p* < .001; baseline versus 5 mg dose: t_[49]_ = −5.653; *p* < .016 Bfc; baseline versus 10 mg dose: t_[49]_ = −6.664; *p* < .016 Bfc; 5 mg dose versus 10 mg dose: t_[49]_ = 2.875; *p* < .016 Bfc) (Fig. 1c).

In parallel, the Profile of Treatment Response (POTR) scale was used to map the medication journey of the patient. The POTR is a clinician-completed scale, used routinely in the CIPPRD as a clinical pharmacovigilance tool. It allows for the assessment and monitoring of a patient over time, and provides clinically meaningful information on the effectiveness of the medication (therapeutic Efficacy Index [EI]), change in symptoms, and side-effects of the medication. The EI is assessed by the degree of medication side effects and the improvement/worsening of symptoms, which are denoted by a score ranging from 0.25 to 4.00. In general, an EI score of >1.5 suggests that the medication is effective over time i.e., is ameliorating the symptoms and improving the overall functioning of the patient without having unacceptable side-effects. In this patient, the POTR revealed the symptom profile of the patient in terms of anxiety, emotional dysregulation, and sleep problems were made much worse following 10 mg propranolol treatment. Moreover, the EI values shifted from 1.00 at 5 mg propranolol to 0.33 following 10 mg propranolol treatment. Collectively, these data indicate that propranolol treatment had no therapeutic effect on the patient and significantly interfered with functioning.

## Conclusions

Here we described a 13 year old girl with RTT who after being prescribed propranolol for 24 h at doses of 5 mg and 10 mg demonstrated dose-dependent paradoxical increases in heart rate. Such an unusual characteristic in a cardiac phenotype following propranolol treatment for managing dysautonomia has not been previously reported in individuals with RTT. Baseline brainstem functions are severely impacted in patients with RTT [23, 25, 32]. As a consequence of this development brainstem immaturity, brainstem autonomic functions can be severely affected. Hitherto the resulting imbalance in the sympathetic and the parasympathetic nervous system (dysautonomia) is a prime suspect in causing cardiac instability and sudden death which accounts for nearly 25% of all deaths in individuals with RTT [15]. In this regard, propranolol would be considered an ideal cardio-protective agent to be used in most patients; however our findings show that in patients with RTT this may not be the case. Evidence from murine studies have indicated that acute treatment with propranolol does not prevent ventricular tachycardia in mice lacking MeCP2 that have an increased susceptibility to ventricular arrhythmias [27]. Moreover, chronic propranolol has no effect on the prolongation of the QTc interval and also failed to prevent cardiac arrhythmias in MeCP2 deficient mice [28]. Coupled with the murine data, collectively the results presented here strongly suggest that caution should be stressed when using propranolol as a treatment to manage dysautonomia in patients with RTT or at the very least its use in RTT patients should be carefully monitored.

Due to the divergent symptomatology in patients with RTT, we cannot rule out the possibility that the paradoxical increase in heart rate following propranolol treatment is caused by a mechanism other than dysautonomia, however, the fact that there were no clinically significant cardiac abnormalities in the patients’ electrocardiogram strongly suggests that these paradoxical increases in heart rate were not due to an inherent cardiac abnormality but rather likely to be governed by a sympathetic-parasympathetic imbalance. As indicated previously [33], one measure of ANS function is respiratory sinus arrhythmia, which in turn is a metric of heart rate variability associated with spontaneous breathing. In terms of neurodevelopmental disorders, the patterns of autonomic dysfunction are not so obvious. For example, respiratory sinus arrhythmia of lower amplitude and faster heart rates has been observed in children with ASD, and it is likely that in these children the vagal brake is less effective and thus the sympathetic influences cannot be sufficiently abrogated [33, 34]). Other studies have explored autonomic dysfunction in RTT [21–23, 25, 32], and have also used heart variability as a marker for autonomic activity in patients with RTT [15]. Recently, an analysis of 132 females with RTT aged between 2 and 43 years from a multi-center study conducted from 1999 to 2012 in four European countries has provided a basis for the dysautonomia in patients with RTT [24]. In this study, all RTT patients had dysautonomia and uncontrolled albeit normal sympathetic tone due to minimal negative feedback of the parasympathetic system, which was the main contributing factor to the dysautonomia seen in these patients [24]. Further prospective studies will be able to shed mechanistic insight into the basis for this dysautonomia that is thought to be unique in patients with RTT [24].

This case report exemplifies this problem and general practitioners and physicians should be cognisant of the problems with the use of propranolol to manage dysautonomia in patients with RTT. As RTT exhibits pleiotropic heterogeneity, it has effects on multiple phenotypic traits that manifest in a wide range of clinical symptoms. In view of this, it is likely that the remodelling of cardiac physiology in individuals with RTT and the resulting interplay between the imbalance between the sympathetic and the parasympathetic nervous system, in particular minimal negative feedback of the parasympathetic branch as suggested recently [24], could account for the paradoxical increase in heart rate observed. A psychophysiological profiling of RTT patients using different medication strategies will be a step forward when exploring treatment options when managing rare disorders such as RTT. This is a case report recording a paradoxical effect on heart rate. A more detailed study looking at larger numbers is warranted.

## Abbreviations

ANS: Autonomic nervous system
ASD: Autism spectrum disorder
CIPPRD: Centre for interventional paediatric psychopharmacology and rare diseases
ECG: Electrocardiogram
EDMS: Effective dosing with minimum side effects
EI: Efficacy index
MeCP2: methyl-CpG binding protein 2
POTR: Profile of treatment response
RTT: Rett syndrome
